# Supplementary material for: Chitosan–Polyethylene Glycol Inspired Polyelectrolyte Complex Hydrogel Templates Favoring NEO-Tissue Formation for Cardiac Tissue Engineering
Source: Gels. 2024 Jan 8;10(1):46. doi: 10.3390/gels10010046 (PMC10815274; doi:10.3390/gels10010046)
Supplement: Supplementary file 1 [file gels-10-00046-s001.zip › Video legends.pdf]

Supplementary Videos

Video S1. Z-stack for the rhodamine phalloidin staining for cell spreading and penetration in 2D control.

Video S2. Z-stack for the rhodamine phalloidin staining for cell spreading and penetration in CP hydrogel.

Video S3. Z-stack for the rhodamine phalloidin staining for cell spreading and penetration in GCP hydrogel.

Video S4. Z-stack for the rhodamine phalloidin staining for cell spreading and penetration in HACP hydrogel.

Video S5. Z-stack for the rhodamine phalloidin staining for cell spreading and penetration in CoCP hydrogel.

Video S6. Z-stack for the rhodamine phalloidin staining for neo-tissue formation by rat LV explants in CP hydrogel.

Video S7. Z-stack for the rhodamine phalloidin staining for neo-tissue formation by rat LV explants in GCP hydrogel.

Video S8. Z-stack for the rhodamine phalloidin staining for neo-tissue formation by rat LV explants in HACP hydrogel.

Video S9. Z-stack for the rhodamine phalloidin staining for neo-tissue formation by rat LV explants in CoCP hydrogel.

Video S10. Z-stack for the rhodamine phalloidin staining for neo-tissue formation by swine LV explants in CP hydrogel.

Video S11. Z-stack for the rhodamine phalloidin staining for neo-tissue formation by swine LV explants in GCP hydrogel.

Video S12. Z-stack for the rhodamine phalloidin staining for neo-tissue formation by swine LV explants in HACP hydrogel.

Video S13. Z-stack for the rhodamine phalloidin staining for neo-tissue formation by swine LV explants in CoCP hydrogel.
